# Supplementary material for: Large scale coherent magnetohydrodynamic oscillations in a sunspot
Source: Nat Commun. 2022 Jan 25;13:479. doi: 10.1038/s41467-022-28136-8 (PMC8789893; doi:10.1038/s41467-022-28136-8)
Supplement: Supplementary file 2 — Description of Additional Supplementary Files [file 41467_2022_28136_MOESM2_ESM.docx]

**Description of Additional Supplementary Files**

**File Name:** Supplementary Video 1

**Description:** Temporal evolution of the observed and reconstructed Doppler velocity.
